# Atrial Cardiomyopathy and Ischemic Stroke: A Monitoring-Integrated Staging and Phenotyping Framework Beyond Atrial Fibrillation

**DOI:** 10.31083/RCM51244

**Published:** 2026-10-13

**Authors:** Ye Cao, Qunxiong Fan, Qing Hao, Yi Feng

**Affiliations:** ^1^Department of Cardiology, Renmin Hospital, Hubei University of Medicine, 442000 Shiyan, Hubei, China; ^2^Department of Pulmonary and Critical Care Medicine, Renmin Hospital, Hubei University of Medicine, 442000 Shiyan, Hubei, China

**Keywords:** atrial cardiomyopathy, atrial fibrillation, ischemic stroke, embolic stroke of undetermined source, secondary prevention, atrial remodeling, thrombosis

## Abstract

In many patients with cryptogenic stroke or embolic stroke of undetermined source (ESUS), the atrium remains clinically relevant even when atrial fibrillation (AF) is not detected with routine or extended cardiac monitoring. The construct of atrial cardiomyopathy (AtCM) addresses this problem by characterizing a measurable atrial substrate rather than relying solely on a binary search for AF. Thus, this narrative review proposes a framework that integrates cardiac monitoring with a staging system to describe AtCM following ischemic stroke. Moreover, this review distinguishes arrhythmogenic AtCM, which primarily facilitates AF detection, from thrombogenic AtCM, an emerging concept that reflects blood stasis, left atrial appendage dysfunction, endocardial–endothelial activation, and coagulation activation. Recent neutral or mixed anticoagulation trials suggest that broad atrial stress markers are insufficient for selecting patients for empiric anticoagulation. Therefore, we present a Stage 0–4 classification system that links phenotype burden to monitoring intensity and research eligibility. This framework is intended to standardize reporting, guide the intensity of monitoring, support upstream risk-factor management, and improve trial enrichment. However, this framework is not intended to replace guideline-based decisions regarding antithrombotic therapy.

## 1. Introduction

Atrial cardiomyopathy (AtCM) has emerged as a clinically relevant atrial disease substrate that may contribute to embolic stroke risk even when atrial fibrillation (AF) is absent, transient, or undetected by conventional monitoring methods [1,2]. The key implication for stroke care is not that every AtCM marker should trigger anticoagulation, but that atrial phenotypes should be interpreted as part of a continuum of substrates with distinct arrhythmogenic and thrombogenic implications.

This distinction has become more important in light of recent embolic stroke of undetermined source (ESUS) and atrial-cardiopathy-enriched trials. Prolonged monitoring increases AF detection after stroke [3,4,5], yet many monitored patients still have no documented AF, and empiric anticoagulation has not outperformed aspirin in unselected ESUS trials [6,7]. The Atrial Cardiopathy and Antithrombotic Drugs In prevention After cryptogenic stroke (ARCADIA) trial further showed that a panel dominated by atrial stress or structural markers did not identify a population in whom apixaban was superior to aspirin [8]. These findings caution against simply counting atrial markers and support a clearer separation between AF-propensity phenotypes and thrombogenic substrate.

Previous reviews have summarized atrial cardiopathy, left atrial (LA) dysfunction, imaging-derived biomarkers, and cryptogenic stroke [9,10,11]. Unlike reviews primarily organized around imaging modalities, AF detection strategies, or general mechanistic frameworks, the present review proposes a monitoring-integrated staging model that distinguishes arrhythmogenic from thrombogenic AtCM. This framework explicitly incorporates trial enrichment concepts and contextualizes the neutral findings of the ARCADIA trial and unselected ESUS anticoagulation trials. Accordingly, we adopt AtCM as the umbrella term, reserve atrial cardiopathy for trial-specific operational definitions, and explicitly label several components as investigational when evidence is insufficient to support routine treatment decisions.

Fig. 1 illustrates the proposed causal network. Risk factors and systemic disease can lead to atrial remodeling, characterized by electrical instability, mechanical dysfunction, stasis, fibrosis, and endocardial-endothelial activation. AF can amplify this pathway, but the atrial substrate itself may also contribute to embolic risk.

**Fig. 1.**
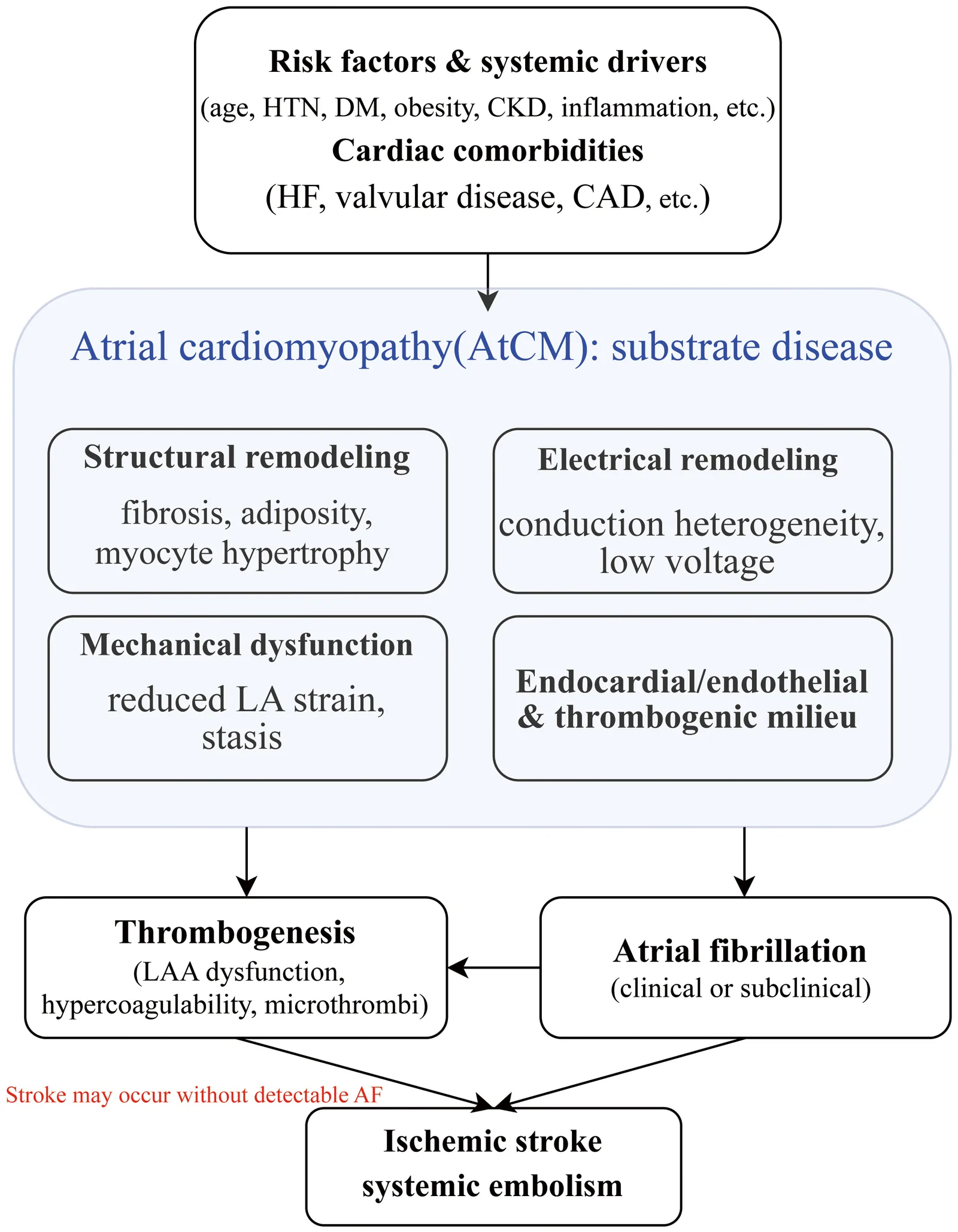
**Atrial cardiomyopathy as a substrate linking atrial remodeling, thrombogenesis, and stroke beyond atrial fibrillation**. Risk factors promote structural remodeling, electrical instability, mechanical dysfunction/stasis, and endocardial-endothelial activation. These processes may support thrombogenesis with or without clinically documented AF. The figure is conceptual and does not imply that any single marker is sufficient to guide anticoagulation. HTN, hypertension; DM, diabetes mellitus; CKD, chronic kidney disease; HF, heart failure; CAD, coronary artery disease; LA, left atrium; LAA, left atrial appendage; AF, atrial fibrillation.

We conducted a focused narrative synthesis by searching PubMed and Embase for articles published between January 2020 and December 2025. Our search used terms including atrial cardiomyopathy, atrial cardiopathy, embolic stroke of undetermined source, left atrial strain, atrial fibrosis, left atrial appendage, and subclinical atrial fibrillation. Foundational studies published before 2020 were included if they defined key concepts, monitoring thresholds, trial context, or operational criteria that remain relevant. In our review, we prioritized consensus updates from post-2021, large prospective cohorts, randomized trials directly related to AtCM-guided strategies, and translational studies focusing on thrombogenesis.

The review proceeds from terminology to application: defining the phenotype, assigning stage, linking stage to rhythm monitoring, and outlining how a more specific definition of thrombogenic substrate could be tested in future studies.

## 2. From Concept to Operation: Definition, Terminology, and Staging

For AtCM to be beneficial after a stroke, three key points should be clearly stated: how it is measured, whether the endpoint is AF detection or embolic risk, and whether the proposed action is part of established care or an investigational approach [1]. Without these distinctions, AtCM markers can be misinterpreted as treatment recommendations or underutilized as monitoring and research tools.

### 2.1 Definition and Endpoint-Specific Nomenclature

We define AtCM as a multidomain atrial substrate that includes structural and fibrotic remodeling, electrical abnormalities, mechanical dysfunction, and a propensity for stasis [1,2,12]. When the endpoint is an embolic stroke rather than AF detection, indicators of endocardial-endothelial or coagulation activation should be considered a thrombogenic subdomain, which remains incompletely standardized for routine clinical decision-making [1,2]. For clarity, we use two endpoint-specific terms. Arrhythmogenic AtCM refers to a substrate that increases the likelihood of AF initiation, maintenance, or recurrence. Thrombogenic AtCM refers to a substrate in which LA/left atrial appendage (LAA) dysfunction, stasis, endocardial activation, or coagulation activation may contribute to embolic risk, even before AF is documented. The second term is employed here as a research and trial-enrichment construct rather than as a stand-alone clinical indication for anticoagulation.

In the literature on stroke trials, the term atrial cardiopathy often refers to a predefined operational panel, such as electrocardiogram (ECG), biomarker, or LA size criteria, used for trial entry [13]. In this review, atrial cardiopathy is used when discussing those trial-defined criteria; AtCM encompasses the broader disease-spectrum concept.

### 2.2 Operational Staging: Rules Before Thresholds

The Stage 0–4 scheme is used here as a descriptive and reporting framework rather than a validated treatment algorithm. Three hierarchy rules are used to improve reproducibility. First, patients should be assigned to the highest stage for which they meet criteria; for example, a patient with both abnormal P-wave terminal force in lead V1 (PTFV1) or another P-wave marker and impaired LA reservoir strain is classified as Stage 2 rather than Stage 1. Second, Stage 4 is defined by documented AF/flutter meeting guideline criteria, overt LA/LAA thrombus, or another established cardioembolic indication, which supersedes the criteria for earlier stages. Third, monitoring intensity should be reported as a stage modifier because the label ‘AF-negative’ has different meanings depending on whether it is based on 24-hour telemetry, 30-day monitoring, or implantable loop recorder (ILR) surveillance.

The thresholds in this framework should be interpreted according to their evidence status. Some cutoffs are practical anchors based on clinical trials or imaging standards, rather than validated, universal AtCM staging criteria. For example, a left atrial volume index (LAVI) >34 mL/m^2^ for LA enlargement follows American Society of Echocardiography/European Association of Cardiovascular Imaging (ASE/EACVI) chamber-quantification standards [14]; PTFV1 >5000 μV·ms and NT-proBNP >250 pg/mL are ARCADIA-derived atrial cardiopathy criteria [13], while this framework uses NT-proBNP 250–1000 pg/mL as a pragmatic early-signal band and treats markedly higher values as a prompt to assess heart failure, renal dysfunction, and acute systemic stress; and LA reservoir strain below the local lower limit of normal, with <23% often used as a practical lower reference boundary, is supported by strain consensus guidance [15]. The lower 20%–23% range should be read as a conservative research range rather than a treatment threshold. Other markers, especially CMR fibrosis/ECV and thrombogenic biomarker panels, should be interpreted using local normative values or prespecified study cutoffs. Monitoring duration is treated as a stage modifier because it changes the meaning of AF-negative status and because prolonged external monitoring and ILR follow-up increase AF detection after cryptogenic stroke [3,4,5,16,17]. Table 1 (Ref. [3,4,5,13,14,15,16,17,18,19]) presents the operational Stage 0–4 framework, including quantitative examples, suggested minimum monitoring, and clinical or research implications.

**Table 1.** **Operational Stage 0–4 framework for AtCM assessment after ischemic stroke**.

Stage	Operational criteria and quantitative examples	Suggested minimum rhythm monitoring	Clinical/research implication and evidence status
0 Exposure/risk drivers	No measurable atrial phenotype. Examples: hypertension, obesity, sleep apnea, diabetes, alcohol exposure, heart failure risk, or vascular risk factors without abnormal ECG, natriuretic peptide, or atrial imaging signal.	Inpatient telemetry and short-duration ECG/Holter monitoring according to local stroke protocols; no minimum prolonged monitoring required solely for Stage 0.	Optimize vascular and atrial risk drivers. This stage is not a justification for anticoagulation or advanced atrial imaging by itself.
1 Early atrial signal	Electrical or biomarker signal without definite mechanical/structural substrate. Examples: PTFV1 >5000 μV·ms or NT-proBNP 250–1000 pg/mL, interpreted with age, renal function, HFpEF, and acute stroke stress; values above this range should prompt evaluation for heart failure, renal dysfunction, or other non-atrial drivers [13]; frequent atrial ectopy may support monitoring escalation but is not a thrombogenic criterion by itself.	At least 30 days of external monitoring in cryptogenic or embolic-appearing stroke [5,17]; consider implantable loop recorder (ILR) when pretest probability is high or recurrent events occur [4,16,17].	Mainly identifies patients in whom AF detection is more likely. This is arrhythmogenic-enrichment territory and does not establish thrombogenic AtCM.
2 Mechanical/structural dysfunction	Definite LA mechanical or structural abnormality. Examples: LAVI >34 mL/m^2^ for LA enlargement [14]; impaired LA reservoir strain <23% or below the local lower limit of normal (vendor-specific; lower values such as <20% increase specificity) [15]; early atrial functional impairment with diastolic disease.	Monitoring beyond 30 days, preferably up to 90 days, or ILR in high-risk patients, especially when stroke pattern, recurrence, or comorbidity profile suggests high probability of occult AF [3,4,5,16,17].	Supports intensified surveillance, standardized LA strain/LAVI reporting, and upstream risk-factor management. Anticoagulation should not be based on this stage alone.
3 Advanced thrombogenic substrate	High-burden substrate with stasis or candidate thrombogenic biology. Examples: LAA sludge/spontaneous echo contrast, very low LAA emptying velocity, extensive atrial fibrosis/ECV, low-voltage substrate, or a prespecified composite combining mechanics/stasis with endothelial/coagulation activation markers.	ILR preferred when available to quantify AF burden and temporal coupling [4,16,17]; TEE/CT/CMR should be used when results may change management or trial eligibility.	Predominantly investigational unless overt thrombus or guideline-defined AF is present [18]. Best suited to mechanism-matched trial enrollment and validation of thrombogenic AtCM composites.
4 Clinical event/actionable diagnosis	Documented AF/flutter meeting guideline criteria, overt LA/LAA thrombus, or another established cardioembolic indication.	As recommended by contemporary stroke and AF guidelines [18,19]; monitoring is used to quantify burden, recurrence, and temporal relation to events rather than to define AtCM alone.	Treat according to established stroke and AF indications, bleeding risk, and shared decision-making [18,19]. LAA strategies require usual guideline indications and patient-level risk assessment.

Threshold anchors include PTFV1 and NT-proBNP levels >250 pg/mL, derived from the ARCADIA trial, whereas the 250–1000 pg/mL range is used here to represent a pragmatic early-signal band. A LAVI >34 mL/m^2^ follows ASE/EACVI chamber-quantification standards. LA reservoir strain <23% is a practical lower reference limit based on strain-imaging consensus guidance, with lower values conferring greater specificity. Monitoring recommendations are extrapolated from post-stroke AF-detection trials and expert consensus documents rather than validated AtCM-staging cutoffs. Quantitative thresholds are provided where evidence supports them. For domains without standardized cutoffs, local normative values or prespecified study thresholds are recommended. PTFV1, P-wave terminal force in lead V1; LAVI, left atrial volume index; LA, left atrium; LAA, left atrial appendage; AtCM, atrial cardiomyopathy; ECG, Electrocardiogram; ILR, implantable loop recorder; TEE, transesophageal echocardiography; ASE, American Society of Echocardiography; EACVI, European Association of Cardiovascular Imaging.

**Supplementary Table 1** translates the Stage 0–4 framework into trial-enrichment questions, research uses, and endpoints. However, it does not function as a clinical treatment algorithm. **Supplementary Table 2** provides a minimum reporting checklist for studies using this framework and should be applied according to the highest stage reported.

### 2.3 Illustrative Application Cases

**Case 1**: An ESUS patient has PTFV1 >5000 μV·ms and NT-proBNP 320 pg/mL, with normal LA size and preserved LA reservoir strain on a standardized echocardiographic protocol. This patient is classified as Stage 1, indicating the need for intensified rhythm monitoring, rather than empiric anticoagulation.

**Case 2**: Another patient with an embolic-appearing stroke exhibits no AF after initial monitoring but has a LAVI of 42 mL/m^2^ and LA reservoir strain below the local lower limit of normal. This patient is categorized as Stage 2, suggesting the need for prolonged monitoring and aggressive upstream risk-factor management, with antithrombotic therapy guided by current guidelines.

**Case 3**: A patient experiences recurrent embolic infarcts and shows significant LAA stasis or sludge on TEE, along with a prespecified endothelial/coagulation activation profile, but no AF is detected after ILR monitoring. This corresponds to a Stage 3 thrombogenic-substrate phenotype. In the absence of overt thrombus or guideline-defined AF, this case is considered investigational and is primarily suitable for trial enrollment rather than routine anticoagulation.

## 3. Measuring AtCM: Phenotypes That Support Staging and Decisions

AtCM should be assessed in a tiered, endpoint-specific manner. The first tier screens broadly for arrhythmogenic AtCM and atrial stress; the second tier refines risk estimation using echocardiographic mechanics and size; the final tier adjudicates potential thrombogenic AtCM with CMR, CT, or TEE when results could change management, monitoring, or trial eligibility. Fig. 2 summarizes this screen-enrich-adjudicate workflow.

**Fig. 2.**
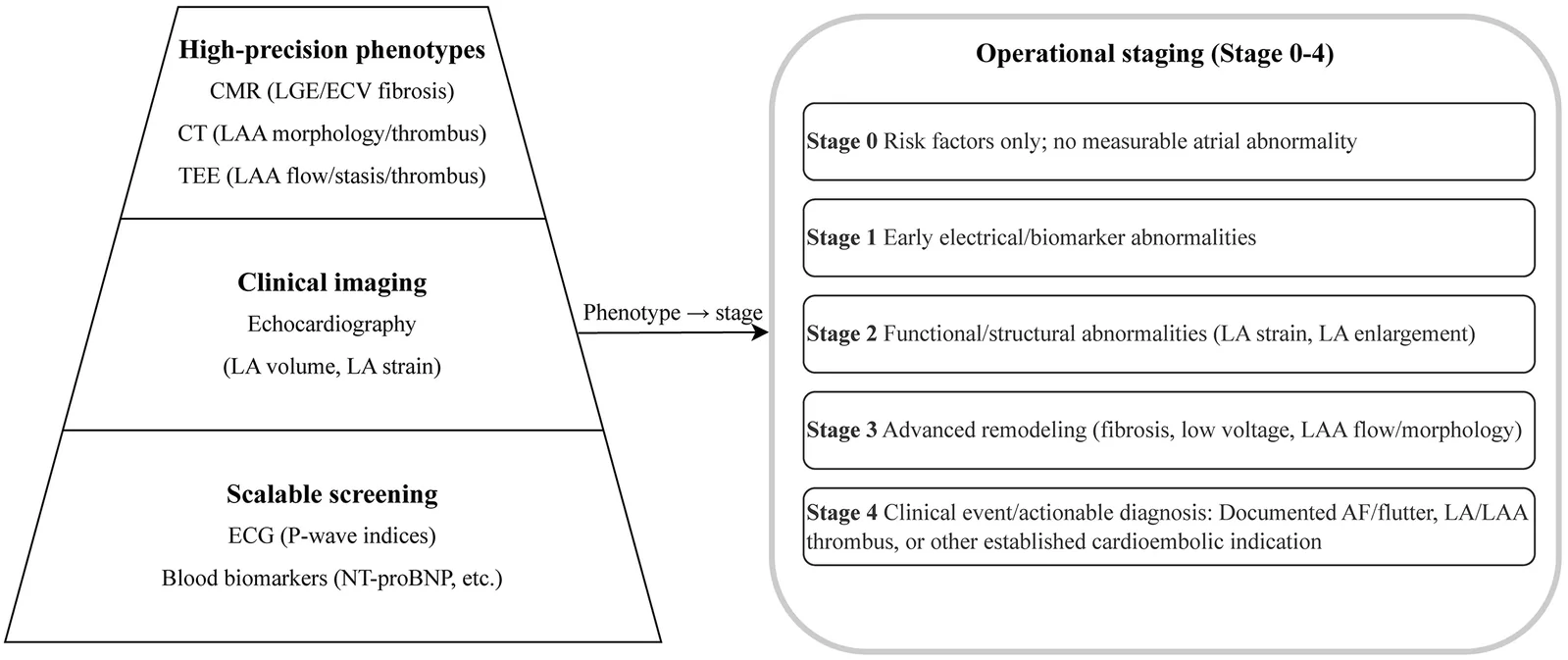
**Tiered phenotyping of atrial cardiomyopathy (AtCM) for stage-based assessment**. Electrocardiogram (ECG) and biomarkers provide broad screening; echocardiographic left atrial (LA) size and mechanics enrich risk assessment; cardiac magnetic resonance (CMR), computed tomography (CT), and transesophageal echocardiography (TEE) adjudicate fibrosis or left atrial appendage (LAA) stasis mechanisms when results may change monitoring, trial eligibility, or management. The pyramid indicates increasing specificity and resource intensity, not increasing certainty that anticoagulation is beneficial.

Screening tests include PTFV1 and other ECG atrial indices, as well as natriuretic peptides. These tests are scalable and useful for monitoring triage, but they often reflect atrial pressure, comorbidity burden, or AF propensity rather than thrombogenicity alone [16,20].

Enrichment tests include LA reservoir strain, LA size, and associated echocardiographic measures. These markers more directly reflect atrial mechanical function and the propensity for stasis [15,21,22,23]. They may therefore contribute to thrombogenic AtCM definitions, but their thresholds depend on the acquisition rhythm, loading conditions, vendor software, segmentation rules, and post-stroke timing.

Adjudication tests include CMR fibrosis/ECV and CT or TEE assessment of LAA morphology, flow, sludge, spontaneous echo contrast, or thrombus. Although these are less scalable, they provide critical tools and information when a thromboembolic mechanism is suspected and should influence monitoring strategy or trial eligibility [24,25,26].

Multidomain composites may offer greater reproducibility than single markers across settings, but external validation remains essential. A practical composite should report at least the electrical/biomarker domain, the LA mechanical/structural domain, rhythm-monitoring exposure, and, when available, the LAA-stasis or thrombogenic-biology domain [13,27,28].

## 4. Core Mechanisms: How a Thrombogenic Substrate May Form

The mechanistic rationale for AtCM-related stroke is clearest when atrial remodeling is linked to its functional consequences. Structural remodeling and fibrosis alter atrial compliance and conduction, impair reservoir function, and promote blood stasis. Inflammatory signaling and endocardial-endothelial injury can then couple this low-flow milieu with coagulation activation. AF may accelerate each step, but it may also be a manifestation of an already diseased atrium rather than the sole causal event in thrombogenesis [29,30,31].

Fibrosis and matrix remodeling are therefore best interpreted as markers of substrate burden, particularly when they co-occur with impaired LA mechanics or LAA dysfunction [22,23,32,33]. Their presence can support mechanistic adjudication, but they should not be treated automatically as modifiable therapeutic targets. Interventions targeting atrial fibrosis or remodeling must demonstrate that modifying the substrate improves embolic, bleeding-balanced, or brain-injury outcomes, rather than relying solely on imaging or electrophysiological surrogates [34,35,36].

Endocardial-endothelial activation and local hypercoagulability provide a plausible link between atrial disease and embolic events in patients without detected AF. However, these markers are not yet standardized for routine clinical use [37]. In this context, thrombogenic AtCM should be viewed as a multidomain pattern rather than a single abnormal test: stasis-prone LA/LAA physiology, mechanical or fibrotic substrate, and endothelial or coagulation activation should be interpreted together. Until such combinations are prospectively validated, they are best used as hypothesis-generating enrichment tools rather than as clinical indications for anticoagulation.

Upstream cardiometabolic and inflammatory exposures should be considered part of the remodeling pathway rather than merely background confounders [18,28]. Obesity, hypertension, sleep apnea, diabetes, systemic inflammation, and HFpEF may shape atrial mechanics and biology over time. The translational question is whether treating these drivers can shift the atrial phenotype and brain-injury outcomes in parallel, even when AF suppression is incomplete.

## 5. Clinical Evidence and Implications: Prediction, Reclassification, and Heterogeneity

### 5.1 Why Do AtCM Markers Predict Stroke Inconsistently?

Observational studies generally support an association between AtCM phenotypes and incident or recurrent embolic events, but effect estimates vary because studies often measure different substrates and use different monitoring protocols [1,22,38]. Functional and mechanical measures, including left atrial strain, have been associated with thromboembolic risk [22,39], while natriuretic peptides have also shown associations with atrial cardiopathy and stroke risk [38]. Fibrosis imaging remains sensitive to acquisition, segmentation, and analysis pipelines [35,40].

A major source of heterogeneity is AF ascertainment. A patient labeled AF-negative after brief inpatient telemetry is not equivalent to one who remains AF-negative after 30-day external monitoring or ILR surveillance. Differences in monitoring intensity affect both the apparent prevalence of arrhythmogenic AtCM and the interpretation of residual embolic risk attributed to the atrial substrate.

Stroke phenotype and timing introduce additional variability. Mixing lacunar, atherosclerotic, and embolic-appearing events dilutes associations that may be specific to an atrial source, while biomarkers and LA loading conditions measured during the acute stroke phase may not reflect the stable substrate. Dichotomizing continuous markers such as NT-proBNP, LAVI, or LA strain can also reduce transportability across cohorts by obscuring risk gradients and masking platform-specific measurement differences.

Finally, comorbidities such as HFpEF, renal dysfunction, hypertension, and systemic inflammation influence both atrial phenotypes and stroke mechanisms. These conditions may strengthen the association between AtCM markers and adverse outcomes, but they can also introduce competing explanations for the observed elevations in biomarkers, atrial enlargement, or brain injury. Therefore, AtCM phenotypes should be interpreted as part of a multidomain pattern that includes monitoring exposure, stroke mechanism, and clinical context, rather than as isolated binary labels.

### 5.2 Incremental Value Beyond Traditional Models

Incremental risk prediction is most defensible when markers are combined across domains rather than interpreted in isolation [38,41,42]. In community cohorts, atrial phenotyping may identify individuals with atrial dysfunction despite modest clinical scores, supporting surveillance and prevention. In ESUS, its current role is to guide monitoring intensity, refine mechanistic classification, and enrich trials; it should not be used as a stand-alone reason for empiric anticoagulation [6,7,8].

### 5.3 Cognition and Covert Brain Injury

AtCM has also been linked to covert brain injury and cognitive outcomes, which may reflect cumulative microembolism, hypoperfusion, or shared vascular vulnerability [43,44,45]. These outcomes may be useful in future trials because they are more frequent than recurrent stroke. However, they still require careful adjudication against small-vessel disease and non-atrial mechanisms.

Taken together, the evidence supports the use of AtCM phenotyping as a framework for risk and mechanism. It does not yet support a simple rule that a single abnormal atrial marker equates to anticoagulation benefit.

## 6. ESUS and Device-Detected Atrial Arrhythmia as Stress Tests

Randomized trials provide the clearest test of whether atrial phenotypes can guide antithrombotic therapy (Table 2, Ref. [6,7,8,46,47]). Unselected ESUS trials showed that empiric anticoagulation without mechanism-specific enrichment is insufficient [6,7]. ARCADIA then showed that atrial cardiopathy, defined by PTFV1, NT-proBNP, or LA enlargement [13], did not identify a group in whom apixaban improved outcomes compared with aspirin [8]. We interpret these findings as evidence that stress-weighted markers may preferentially enrich for AF propensity or comorbidity rather than a directly thrombogenic atrial substrate. The appropriate response is not empiric anticoagulation, but more specific enrichment in future trials.

**Table 2.** **Randomized trials informing anticoagulation and atrial-substrate enrichment**.

Trial	Enrichment strategy tested	Comparison	Core mechanistic lesson
NAVIGATE ESUS [6]	No atrial or mechanistic enrichment (broad ESUS)	Rivaroxaban vs aspirin	Empiric anticoagulation without substrate definition fails; bleeding excess highlights the cost of mechanism-agnostic treatment.
RE-SPECT ESUS [7]	No atrial or mechanistic enrichment (broad ESUS)	Dabigatran vs aspirin	Neutral effect reinforces etiologic heterogeneity and the need for targeted substrate enrichment.
ARCADIA [8]	ECG- and biomarker-based atrial cardiopathy (without AF)	Apixaban vs aspirin	Neutral result suggests current atrial cardiopathy panels preferentially capture atrial stretch or AF propensity rather than a truly thrombogenic substrate.
ARTESiA [46]	Device-detected subclinical AF (AHRE)	Apixaban vs aspirin	Stroke reduction offset by increased bleeding; benefit appears risk- and burden-dependent, supporting phenotype-conditioned treatment thresholds.
NOAH-AFNET 6 [47]	AHRE without ECG-documented AF	Edoxaban vs placebo/aspirin	Absence of net benefit underscores the risk of extrapolating anticoagulation without confirming causal thromboembolic mechanisms.

Abbreviations: AF, atrial fibrillation; AHRE, atrial high-rate episode; AtCM, atrial cardiomyopathy; ESUS, embolic stroke of undetermined source; ECG, electrocardiogram; LA, left atrium; NT-proBNP, N-terminal pro-B-type natriuretic peptide; PTFV1, P-wave terminal force in lead V1; SCAF, subclinical atrial fibrillation.

Monitoring intensity is not merely a background detail. The more intensively patients are monitored, the more AF is detected. However, the clinical significance of detected AF depends on AF burden, episode duration, temporal proximity to the index stroke, and the underlying atrial substrate [17,48,49]. For this reason, AF-negative status should be defined by the monitoring protocol, and studies should report AF burden rather than reporting only incident AF.

Device-detected AHRE and SCAF trials further show that the benefits of detection and treatment are not identical [46,47]. In general, anticoagulation is most plausible when atrial arrhythmia occurs in a high-risk patient with a substrate consistent with thromboembolism, while bleeding risk remains clinically important [46,47,50,51,52].

For future trials, we propose multidomain enrichment that combines LA/LAA mechanics or stasis, structural substrate, prespecified rhythm monitoring, and candidate thrombogenic biology. This is a research strategy, not a current clinical treatment rule. Fig. 3 summarizes this trial-enrichment strategy.

**Fig. 3.**
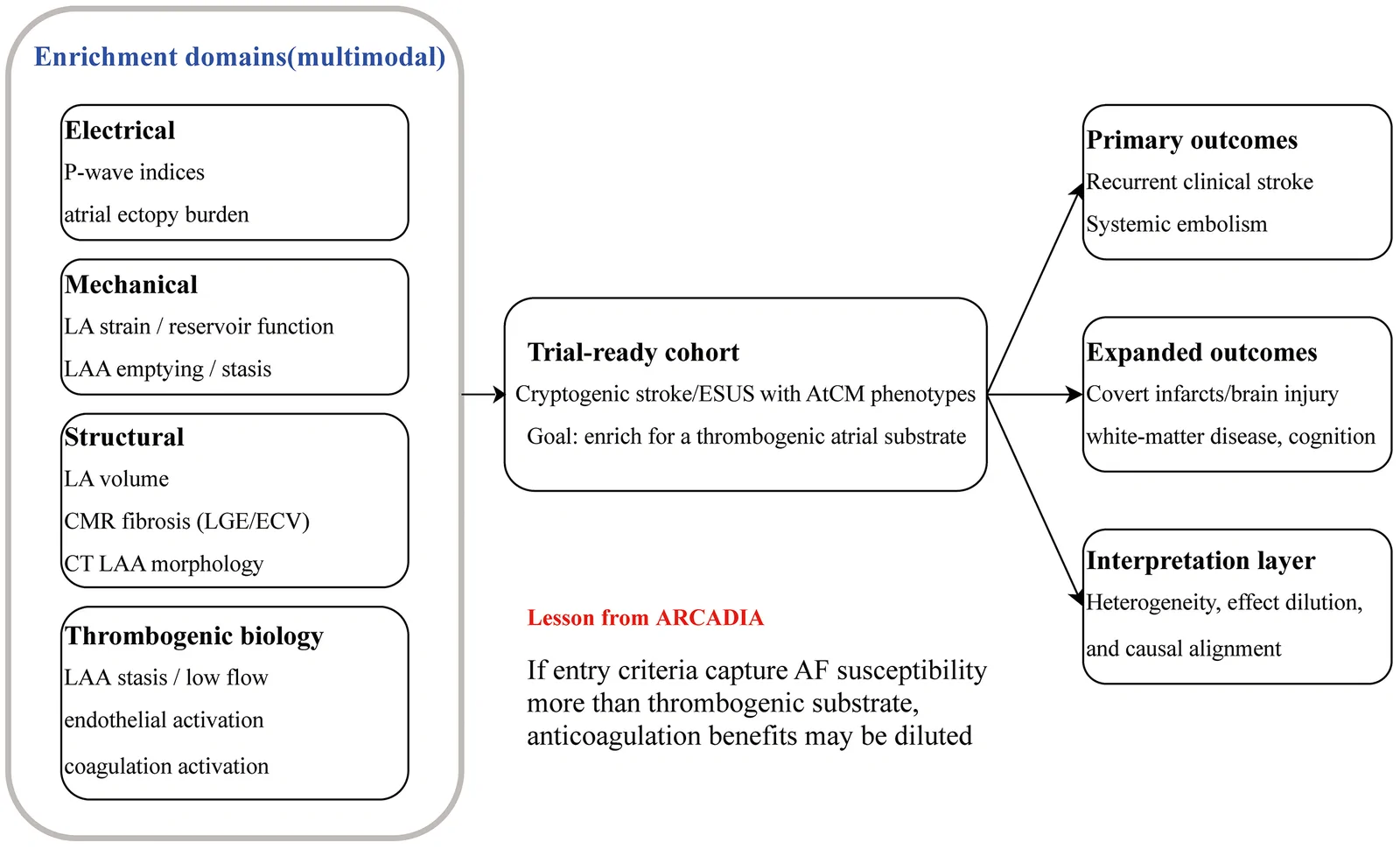
**Enrichment framework for future atrial cardiomyopathy (AtCM)-stroke trials**. Broad definitions of embolic stroke of undetermined source (ESUS) dilute treatment effects because competing mechanisms are common. A more focused design would prespecify rhythm monitoring, require mechanical/stasis or structural evidence of atrial disease, and evaluate candidate thrombogenic biology. AF-negative, no documented atrial fibrillation; LAA, left atrial appendage; CMR, cardiac magnetic resonance; CT, computed tomography; TEE, transesophageal echocardiography. This framework is intended for trial design and does not replace guideline-based treatment indications.

## 7. A Clinical Decision-Support Pathway, Not a Treatment Mandate

Fig. 4 integrates staging, phenotyping, and monitoring into a practical pathway. The pathway includes three safeguards. First, it supports decisions on the intensity of evaluation and monitoring rather than directly prescribing anticoagulation. Second, it distinguishes established indications, such as documented AF or thrombus, from investigational enrichment phenotypes. Third, it requires clinicians to reassess non-atrial mechanisms of stroke before attributing recurrence or covert injury to AtCM. Decisions about anticoagulation, left atrial appendage occlusion, and secondary stroke prevention should remain anchored in contemporary AF and stroke-prevention guidelines [18,19].

**Fig. 4.**
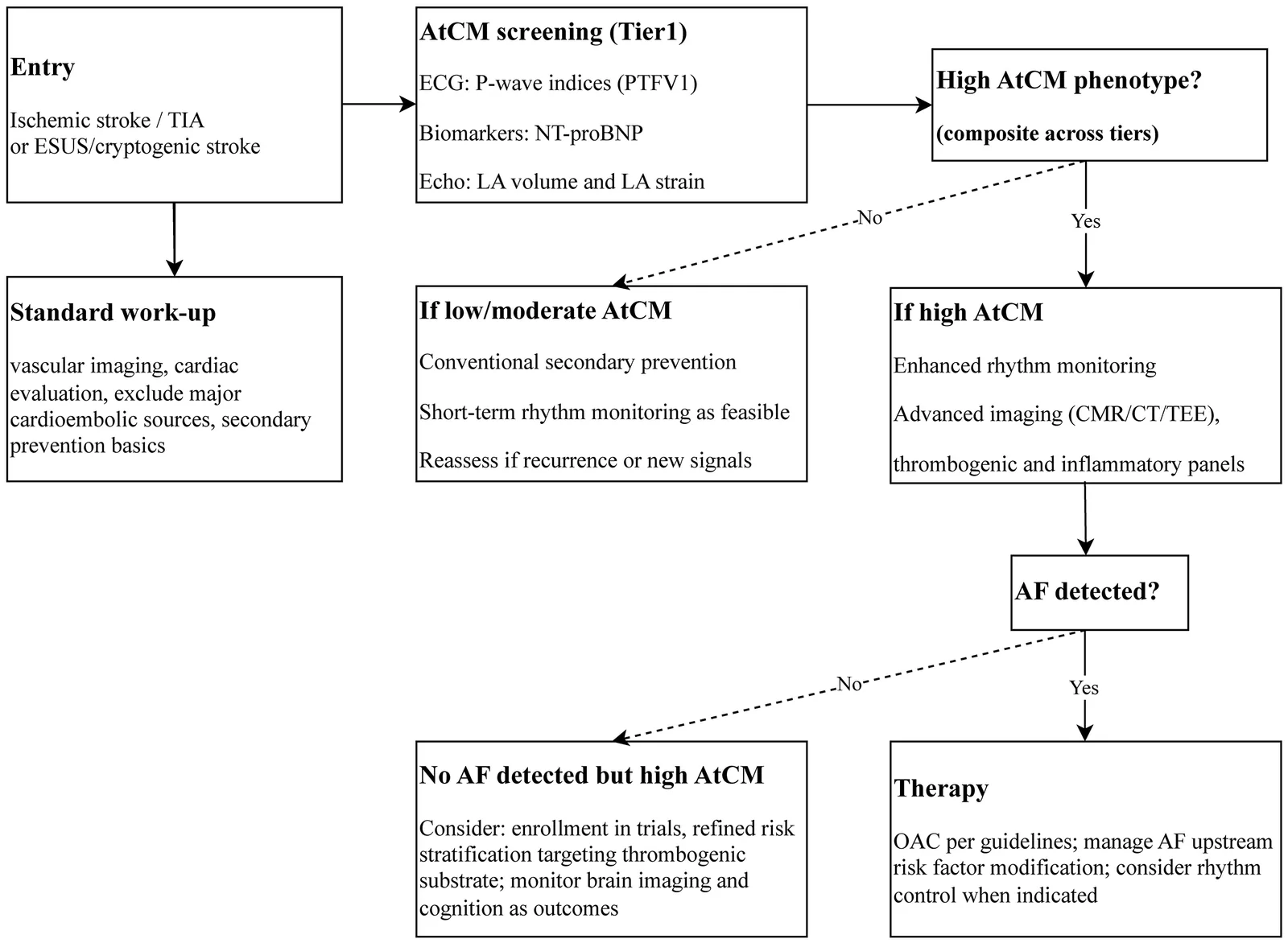
**Monitoring-integrated pathway for atrial cardiomyopathy (AtCM) assessment after ischemic stroke**. Initial electrocardiogram (ECG), biomarkers and a standard stroke workup identify patients for echocardiographic LA assessment. Abnormal mechanical or structural phenotypes warrant longer rhythm monitoring and upstream risk-factor treatment. Advanced imaging or LAA assessment by cardiac magnetic resonance (CMR), computed tomography (CT), or transesophageal echocardiography (TEE) is reserved for selected patients in whom the result may change management or trial eligibility. Anticoagulation remains guided by established indications unless future trials validate a thrombogenic AtCM treatment strategy.

## 8. Future Agenda: Validation Before Implementation

Validate Stage 0–4 assignment in prospective stroke cohorts with prespecified monitoring duration and blinded stroke-mechanism adjudication.

Develop and externally validate thrombogenic AtCM composites that include LA/LAA stasis, mechanical dysfunction, structural remodeling, and endothelial and coagulation activation.

Standardize acquisition and reporting for LA strain, LAVI, CMR fibrosis/ECV, LAA flow, biomarker timing, and rhythm-monitoring burden.

Use continuous or protocolized rhythm monitoring to distinguish AF propensity from AF-independent thromboembolic substrate.

Broaden endpoints to include recurrent embolic stroke, covert infarction, progression of vascular brain injury, cognition, bleeding, and net clinical benefit.

Test mechanism-matched strategies, including precision antithrombotic therapy, upstream risk-factor reversal, and LAA-centered approaches, only in populations enriched for the relevant mechanism.

Two practical next steps would be particularly useful: a multicenter AtCM-stroke registry with harmonized echo/CMR/TEE protocols, longitudinal biospecimens, and standardized rhythm monitoring; and an imaging-stasis consortium that provides shared analysis code, quality-control benchmarks, and transportable cutoffs for LA strain, LAA stasis, and fibrosis quantification.

## 9. Conclusions

AtCM is best understood as a measurable atrial disease substrate that can be arrhythmogenic, thrombogenic, or both. The proposed Stage 0–4 framework aims to make this substrate easier to describe, compare, and test after ischemic stroke by combining phenotype burden with explicit monitoring intensity. Current evidence supports AtCM phenotyping for risk stratification, monitoring allocation, mechanism adjudication, and trial enrichment. It does not yet support routine anticoagulation based solely on isolated atrial stress or structural markers in the absence of AF, thrombus, or another established indication. The next step for the field is prospective validation of multidomain thrombogenic AtCM definitions linked to treatment benefit and net clinical outcome.

## Abbreviations

AF, atrial fibrillation; AHRE, atrial high-rate episode; AtCM, atrial cardiomyopathy; CMR, cardiac magnetic resonance; CT, computed tomography; ECG, electrocardiogram; ECV, extracellular volume; ESUS, embolic stroke of undetermined source; HFpEF, heart failure with preserved ejection fraction; ILR, implantable loop recorder; LA, left atrium; LAA, left atrial appendage; LAVI, left atrial volume index; LGE, late gadolinium enhancement; NOAC, non-vitamin K oral anticoagulant; NT-proBNP, N-terminal pro-B-type natriuretic peptide; PTFV1, P-wave terminal force in lead V1; SCAF, subclinical atrial fibrillation; TEE, transesophageal echocardiography.
