# Supplementary material for: Atrial Cardiomyopathy and Ischemic Stroke: A Monitoring-Integrated Staging and Phenotyping Framework Beyond Atrial Fibrillation
Source: Rev Cardiovasc Med. 2026 Oct 13;27(10):51244. doi: 10.31083/RCM51244 (PMC13732666; doi:10.31083/RCM51244)
Supplement: Supplementary file 1 [file 2153-8174-27-10-51244-s1.zip › Supplementary Material.docx]

Supplementary Table 1. Investigational trial-enrichment implications of the Stage 0-4 atrial cardiomyopathy (AtCM) framework.

Supplementary Table 1 is intended for research design and trial enrichment, not as a clinical treatment algorithm. Clinical management should follow established stroke and atrial fibrillation (AF) guidelines.

| **Stage** | **Main research uncertainty** | **Trial-enrichment use** | **Endpoint / caution** |
| --- | --- | --- | --- |
| 0 | Which risk-factor profiles progress to measurable AtCM? | Prevention cohorts or registry follow-up, not anticoagulation trials. | Incident AtCM phenotype, AF detection, vascular risk control. Avoid treating exposure alone as atrial-source stroke. |
| 1 | Does an electrical/biomarker signal mainly predict AF detection or thrombogenic biology? | Monitoring-yield studies, AF prediction models, and biomarker trajectory studies. | AF or subclinical atrial fibrillation (SCAF) detection, AF burden, N-terminal pro-B-type natriuretic peptide (NT-proBNP) or P-wave terminal force in lead V1 (PTFV1) change. Avoid interpreting isolated stress markers as anticoagulation indications. |
| 2 | Does mechanical/structural dysfunction improve risk reclassification beyond clinical scores and ECG/biomarkers? | Risk-stratification studies and upstream-intervention trials. | Recurrent embolic events, covert infarcts, LA strain improvement, AF burden. Avoid non-prespecified strain thresholds. |
| 3 | Can a validated thrombogenic substrate identify patients with net benefit from antithrombotic or left atrial appendage (LAA)-centered strategies? | Mechanism-matched trials requiring stasis/LAA dysfunction plus structural/mechanical and biology markers. | Recurrent embolic stroke, covert infarcts, bleeding, net clinical benefit. Avoid routine anticoagulation before prospective validation unless AF or thrombus or another guideline indication is present. |
| 4 | How should established indications be integrated with substrate burden and bleeding risk? | Guideline-based treatment studies and registries evaluating AF burden, thrombus, recurrence, and net clinical benefit. | Clinical stroke, systemic embolism, bleeding, mortality, cognition, MRI outcomes. Avoid ignoring competing stroke mechanisms once AF is detected. |

Supplementary Table 2. Minimum reporting checklist for atrial cardiomyopathy (AtCM) phenotyping studies, linked to staging domains.

Supplementary Table 2 is intended as a reporting checklist for studies using the Stage 0-4 framework. It should be applied according to the highest stage reported: Stage 1 studies should at minimum specify electrocardiogram (ECG)/biomarker and monitoring methods; Stage 2 studies should specify left atrial (LA) mechanical and structural methods; Stage 3 studies should additionally report left atrial appendage (LAA)-stasis, cardiac magnetic resonance (CMR), transesophageal echocardiography (TEE), computed tomography (CT), or thrombogenic-biology methods when used.

| **Domain** | **Minimum to report** | **Preferred/advanced elements** | **Common pitfalls** | **Why it matters (comparability/actionability)** |
| --- | --- | --- | --- | --- |
| Electrocardiogram (ECG; conduction) | Acquisition settings; lead set; automated vs manual P-wave metrics; continuous vs threshold | Serial ECG trajectories; signal-averaged ECG; device-derived atrial ectopy | Pipeline/vendor drift; post-stroke acute effects; dichotomization | Enables reproducible screening and monitoring triage |
| Echocardiography (mechanics) | Rhythm at acquisition; reservoir strain; frame rate; vendor/software; segmentation rules | Conduit/booster strain; left atrial (LA) stiffness surrogates; left atrial appendage (LAA) Doppler when available | Non-comparable strain definitions; inconsistent segmentation; load dependence | Bridges substrate to risk and guides escalation (monitoring/imaging) |
| Cardiac magnetic resonance (CMR) / computed tomography (CT) (structure/left atrial appendage [LAA]) | Late gadolinium enhancement (LGE) / extracellular volume (ECV) protocol (if used); segmentation; LAA morphology/flow metrics; quality control | Repeat imaging for trajectories; combined voltage-imaging mapping | Treating prognostic markers as procedural targets; selection bias | Aligns phenotypes with mechanisms; supports adjudication and enrichment |
| Biomarkers (biology) | Timing relative to event; assay; continuous values; renal/HF context | Paneling (N-terminal pro-B-type natriuretic peptide (NT-proBNP) + coagulation/inflammation markers); repeated measures | Threshold-only reporting; confounding by left ventricular (LV)/renal disease | Stabilizes phenotyping across centers; enables gradients and interactions |
| Rhythm monitoring | Tool; duration; timing; atrial fibrillation (AF) burden definition; adjudication | Continuous monitoring; standardized reporting of AF detected after stroke time windows | Variable intensity leads to biased "AF-independent" claims | Key for separating causal AF vs marker AF and interpreting trials |
| Stroke phenotyping | Central adjudication; embolic vs lacunar classification; imaging patterns | Covert infarcts; MRI small vessel disease markers | Endpoint heterogeneity dilutes treatment effects | Improves mechanism matching and endpoint sensitivity |
| Treatment exposure/background care | Antithrombotic regimen; risk factor control; statin/blood pressure (BP) status | Time-varying exposures; adherence measures | Unmeasured co-interventions | Necessary to interpret incremental benefit and neutrality |

Abbreviations used in the supplementary tables: AF, atrial fibrillation; AtCM, atrial cardiomyopathy; BP, blood pressure; CMR, cardiac magnetic resonance; CT, computed tomography; ECG, electrocardiogram; ECV, extracellular volume; HF, heart failure; HFpEF, heart failure with preserved ejection fraction; ILR, implantable loop recorder; LA, left atrium; LAA, left atrial appendage; LAVI, left atrial volume index; LGE, late gadolinium enhancement; LV, left ventricular; MRI, magnetic resonance imaging; NT-proBNP, N-terminal pro-B-type natriuretic peptide; PTFV1, P-wave terminal force in lead V1; SCAF, subclinical atrial fibrillation; TEE, transesophageal echocardiography.
